# Hypoxia decreases creatine uptake in cardiomyocytes, while creatine supplementation enhances HIF activation

**DOI:** 10.14814/phy2.13382

**Published:** 2017-08-21

**Authors:** Lucia Santacruz, Antonio Jose Luis Arciniegas, Marcus Darrabie, Jose G. Mantilla, Rebecca M. Baron, Dawn E. Bowles, Rajashree Mishra, Danny O. Jacobs

**Affiliations:** ^1^ Department of Molecular Biology and Biochemistry The University of Texas Medical Branch Galveston Texas; ^2^ Department of Natural Sciences Bowie State University Bowie Maryland; ^3^ Department of Medicine Pulmonary and Critical Care Division Brigham and Women's Hospital Harvard Medical School Boston Massachusetts; ^4^ Duke University Medical Center Durham North Carolina; ^5^ Department of Surgery The University of Texas Medical Branch Galveston Texas; ^6^ Institute for Translational Sciences University of Texas Medical Branch Galveston Texas

**Keywords:** AMP‐activated kinase, cardiac metabolism, creatine, hypoxia adaptation, membrane transport

## Abstract

Creatine (Cr), phosphocreatine (PCr), and creatine kinases (CK) comprise an energy shuttle linking ATP production in mitochondria with cellular consumption sites. Myocytes cannot synthesize Cr: these cells depend on uptake across the cell membrane by a specialized creatine transporter (CrT) to maintain intracellular Cr levels. Hypoxia interferes with energy metabolism, including the activity of the creatine energy shuttle, and therefore affects intracellular ATP and PCr levels. Here, we report that exposing cultured cardiomyocytes to low oxygen levels rapidly diminishes Cr transport by decreasing *V*_max_ and *K*_m_. Pharmacological activation of AMP‐activated kinase (AMPK) abrogated the reduction in Cr transport caused by hypoxia. Cr supplementation increases ATP and PCr content in cardiomyocytes subjected to hypoxia, while also significantly augmenting the cellular adaptive response to hypoxia mediated by HIF‐1 activation. Our results indicate that: (1) hypoxia reduces Cr transport in cardiomyocytes in culture, (2) the cytoprotective effects of Cr supplementation are related to enhanced adaptive physiological responses to hypoxia mediated by HIF‐1, and (3) Cr supplementation increases the cellular ATP and PCr content in RNCMs exposed to hypoxia.

## Introduction

Hypoxia plays a key role in the pathogenesis of coronary artery disease, stroke, and peripheral artery disease (Michiels 2004). Exposure to low oxygen content alters many systems that are needed for energy homeostasis: oxidative phosphorylation is inhibited, and creatine kinase activity and intracellular creatine (Cr), phosphocreatine (PCr), and ATP levels are decreased (Jennings and Reimer 1991). Profound alterations in Cr and PCr levels are consistently observed in heart failure (Neubauer 2007) and in the peri‐infarct region of the heart in animal models of ischemic injury (Hu et al. 2006). Cardiomyocytes cannot synthesize Cr and depend on transport across the cell membrane to maintain intracellular Cr levels. Cr transport is provided by a membrane protein, the creatine transporter (CrT), which belongs to the SLC6 gene family of transporters (Nash et al. 1994; Snow and Murphy 2001). CrT is a symporter that uses the energy accumulated in the sodium gradient across the membrane to drive the “uphill” transport of Cr into the cell. During the transport cycle, a Cl^−^ ion is also translocated into the cell with a stoichiometry of 2Na^+^:1Cl^−^:1Cr (Wyss and Kaddurah‐Daouk 2000). Homeostasis of intracellular Cr content is therefore controlled by modulation of Cr transport capacity. In cardiac and skeletal myocytes, Cr uptake is affected by the extracellular Cr concentration: increases in extracellular Cr content decrease Cr uptake (Loike et al. 1988; Darrabie et al. 2011), whereas decreases in Cr content increase Cr uptake (Loike et al. 1988; Darrabie et al. 2011). These changes in Cr transport capacity are secondary to an increase or decrease in *V*_max_, with no alteration in *K*_m_. Recently, we (Darrabie et al. 2011) and others (Li et al. 2010) have demonstrated that activation of AMP‐activated kinase (AMPK), the cell's energy master regulator (Hardie et al. 2012), modulates Cr transport in a tissue‐specific manner. AMPK activation increases *V*_max_ of Cr transport in cardiomyocytes in culture, whereas AMPK activation in a kidney cell line decreases Cr transport. In cardiomyocytes, these changes in *V*_max_ correlate with changes in abundance of the cell‐surface fraction of CrT protein, indicating that changes in the cell‐surface population are associated with the cellular responses to extracellular Cr availability. In the heart, AMPK is activated by hypoxia, upregulating energy‐producing pathways and decreasing energy expenditure (Kemp et al. 1999; Dyck and Lopaschuk 2006). AMPK activation also appears to have a protective effect in animal models of cardiac ischemia (Borger et al. 2008; Kim et al. 2011; Li et al. 2013).

Changes in the expression of hypoxia‐inducible factor‐1 (HIF‐1), a transcription factor required for cellular adaptation to low‐oxygen conditions, are also likely relevant. HIF‐1 is composed of *α* and *β* subunits; HIF‐1*β* levels are not altered by normoxia, whereas HIF‐1*α* is normally ubiquitinated and targeted for proteosomal degradation. During hypoxia, HIF‐1*α* is no longer targeted for degradation and forms a dimer with HIF‐1*β*, becoming a transcription factor that interacts with hypoxia‐responsive elements (HREs), upregulating the expression of genes such as VEGF, NOS2, adrenomedullin, endothelin 1, erythropoietin, GLUT1, and GLUT3, among others (Wenger 2000; Su et al. 2002; Sharp and Bernaudin 2004; Ziello et al. 2007). Together, the activation of AMPK and HIF‐1 signaling pathways are thought to constitute a functional axis critical for the survival of cell exposed to hypoxia (Wang et al. 2012).

The benefits of Cr supplementation in the presence of oxidative stress, a hallmark of tissues subjected to hypoxia, have been demonstrated in vitro and in animal models. For example, C2C12 myoblasts supplemented with Cr and challenged with H_2_O_2_ were capable of differentiating into myotubes, whereas nonsupplemented H_2_O_2_‐treated C_2_C_12_ were not (Sestili et al. 2009). Similarly, protective effects against oxidative stress were observed in spinal cord neuroblast cell cultures supplemented with Cr (Sestili et al. 2009) and in rodent models of cerebral ischemia (Zhu et al. 2004; Prass et al. 2007; Perasso et al. 2013), stroke (Prass et al. 2007), Huntington's disease (Dedeoglu et al. 2003), and fetal hypoxic stress (Ireland et al. 2011; Dickinson et al. 2014). Recently, it was reported that an increase in cardiac Cr content exerted a protective effect in a rodent model of ischemia and reperfusion (Lygate et al. 2012). Finally, we have demonstrated that Cr supplementation ameliorates oxidative stress caused by doxorubicin in cultured cardiomyocytes (Santacruz et al. 2015).

In this study, we analyzed the effects of hypoxia on CrT transport kinetics, which has not been reported previously. We tested the hypothesis that hypoxia decreases CrT function. Rat neonatal cardiomyocyte (RNCM) cultures were used in a validated in vitro experimental design that recapitulates ischemia‐induced hypoxia. We also evaluated the effect of Cr supplementation on HIF‐1 activation and cell survival. We report that exposure of cardiomyocytes in culture to low oxygen rapidly diminishes Cr transport by decreasing *V*_max_ and *K*_m_. Cr supplementation significantly increases ATP and PCr content and augments HIF‐1 activation, thereby increasing the cellular adaptive response to hypoxia.

## Materials and Methods

### Cell culture and hypoxia model validation

RNCMs were harvested from 2‐day‐old Sprague Dawley pups as described previously (Bursac et al. 1999; Santacruz et al. 2015) following protocols approved by the Institutional Animal Care and Use Committees at Duke University, Harvard Medical School and Brigham Women's Hospital. Hypoxia was achieved by placing the cells in a hypoxia incubator chamber (STEMCELL Technologies, Vancouver, BC) filled with a 5% CO_2_‐nitrogen balanced gas mixture for 5 min at 15 L/min (final oxygen < 1% inside the chamber). The experimental model was validated by quantifying HIF‐1‐mediated transcriptional activity measured using the HRE luciferase reporter gene as described previously (Lima et al. 2009). RNCMs were transduced with HRE adenovirus (HRE‐Ad, 200 viral particles/cell). Forty‐eight hours after transduction, the cells were incubated in hypoxic conditions for varying times (2–24 h as noted in figure legends). HIF‐1 activation was determined by measuring luciferase expression using the luciferase assay system (Promega, Madison WI). As per experimental design, cultures were supplemented for 24 h with 1 mmol/L Cr and/or 0.5 mmol/L AICAR before a 12‐h incubation in hypoxic conditions, where supplementation was continued.

### CrT‐AAV preparation and transduction of RNCM cells

RNCMs have low Cr transport; therefore, to facilitate the kinetic and biochemical analysis of Cr transport in RNCMs exposed to hypoxia, an adeno‐associated virus encoding the human isoform of CrT was prepared by excising the CrT open reading frame from a pcDNA 3.1 construct described previously (Darrabie et al. 2012) using *EcoR*I and *Hind*III restriction enzymes and ligated into the pTR vector. Recombinant CrT‐AAV was generated by standard triple transfection method (Grieger et al. 2006) using XX6‐80 helper plasmid with the SASTG packaging plasmid (Piacentino et al. 2012) and the CrT‐TR plasmid. Recombinant SASTG‐CrT was purified and viral titer was evaluated by dot‐blot hybridization (Grieger et al. 2006). Experimentation began 48 h after cells were transduced with 3000 viral particles/cell.

### Immunoblots

RNCMs were cultured in six‐well plates. Cells were scraped in ice‐cold lysis buffer (150 mmol/L NaCl, 1% Triton X‐100, 50 mmol/L Tris‐HCl, pH 7.4) containing protease inhibitors (mini‐Complete protease inhibitors, Roche Indianapolis, IN) and phosphatase inhibitors (5 mmol/L NaF, 1 mmol/L phenylmethylsulfonylfluoride, 2.5 mmol/L Na_2_P_2_O_7_, 50 mmol/L *β*‐Glycerol, 1 mmol/L Na_3_VO_3,_ Sigma St Louis, MO). Cell lysates were centrifuged at 4°C for 30 min at 100,000*g*. The supernatant protein concentration was determined by bicinchoninic acid (BCA) protein assay (Pierce Biotechnology). Supernatants were mixed with sample buffer (125 mmol/L Tris‐HCl, pH 6.8, 20% glycerol, 6% sodium dodecyl sulfate, 10% *β*‐mercaptoethanol) and then boiled for 3‐5 min. Equal amounts of protein were subjected to SDS‐PAGE and transferred to nitrocellulose or PVDF membranes. For AMPK detection, membranes were probed with rabbit anti‐alpha AMPK antibodies to detect total AMPK and rabbit anti‐AMPK phosphothreonine (Thr‐172) to detect activated AMPK, both at a 1:1000 dilution. Phospho‐acetyl‐CoA carboxylase (pACC; a downstream target of AMPK) was detected with 1:100 dilutions of rabbit anti‐phospho‐ACC. The secondary antibody was HRP‐conjugated goat anti‐rabbit at a 1:10,000 dilution. TATA box binding protein was used as a loading control on immunoblots. It was detected using a 1:1000 dilution of mouse monoclonal antibody and purchased from AbCam (Cambridge, MA). All other primary antibodies were purchased form Cell Signaling Technology (Danvers, MA). Bands were visualized using an ECL Western blotting detection system GE Healthcare (Piscataway, NJ) and quantified by densitometry using the ImageJ algorithm (National Institutes of Health, Bethesda).

### Apoptosis and cellular viability assays

Cell viability was measured using the luminescent CellTiter‐Glo Viability Assay (Promega, Madisom WI). Apoptosis was quantified by measuring the activity of Caspase 3/7 using the Caspase‐Glo 3/7 kit (Promega Madison, WI).

### ^14^C‐Cr transport measurements in RNCMs

^14^C‐Cr uptake was measured in triplicate as described previously (Darrabie et al. 2011). Cells were washed twice with choline buffer (150 mmol/L choline chloride; 1 mmol/L CaCl_2_; 5 mmol/L MgCl_2_; 2 mmol/L KCl; 5 mmol/L HEPES‐Tris, pH 7.5) at room temperature, followed by a 10‐min incubation at 37°C in a humidified 5% CO_2_ incubator in sodium uptake buffer (150 mmol/L sodium chloride; 1 mmol/L CaCl_2_; 5 mmol/L MgCl_2_; 2 mmol/L KCl; 5 mmol/L HEPES‐Tris, pH 7.5) radiolabeled with 0.550 *μ*Ci/mL for RNCM ^14^C‐Cr (55 mCi/mmol; American Radiolabeled Chemicals, St. Louis, MO), with final Cr concentration for time course experiments adjusted to 15 *μ*mol/L using a nonradiolabeled compound. Uptake was terminated by aspirating the radiolabeled solution, followed by three washes with ice‐cold choline buffer. The cells were lysed in 0.5 mL of 500 mmol/L NaOH and heated to 80°C for 30 min. A 100 *μ*L aliquot of lysed cells was subjected to scintillation counting in a Beckman Coulter LS 6500 liquid scintillation counter. Cr transport was normalized to protein concentration. Cr uptake data were expressed as nanomoles per milligram of protein. Protein concentration was determined using the bicinchoninic acid protein assay (Pierce Biotechnology) and bovine serum albumin as a standard. For kinetic analysis, cells were grown in 24‐well plates and transduced as described earlier. During the uptake assays, the extracellular Cr concentrations ranged from 5 *μ*mol/L to 305 *μ*mol/L as described earlier. The number of independent experiments used for data analysis is noted in the corresponding figure legend. Michaelis–Menten plots were generated using curve‐fitting software (SigmaPlot^®^ ver. 9.0).

### Quantitation of Cr, PCr, and ATP

Cellular Cr content was quantified using colorimetric assay (BioVision, Milpitas, CA). RNCMs were cultured in 24‐well plates as described earlier. Cr content was measured following the manufacturer's protocols and expressed relative to protein content. ATP and PCr content were measured as described by Loo et al. (Wibom et al. 1991; Loo et al. 2015) in RNCMs grown in 24‐well plates as described earlier. ATP was measured using Lonza's ViaLight plus kit (Salisbury, MD). PCr was determined using a luminometric assay in perchloric acid cellular extracts following the addition of creatine kinase and ADP as described in Wibom et al. (1991). Data reported are the mean ± standard error of the mean (SEM), of triplicates, obtained from three independent experiments, and normalized to control values.

### Statistical analysis

Data are reported as mean ± SEM. Data were analyzed using *T*‐test, ANOVA, or two‐way ANOVA as appropriate. Tukey's test was used for post hoc, pairwise, intergroup comparisons (Prism 7) where indicated. Alpha error values <0.05 were considered significant.

## Results

### Model validation

**Figure 1:**
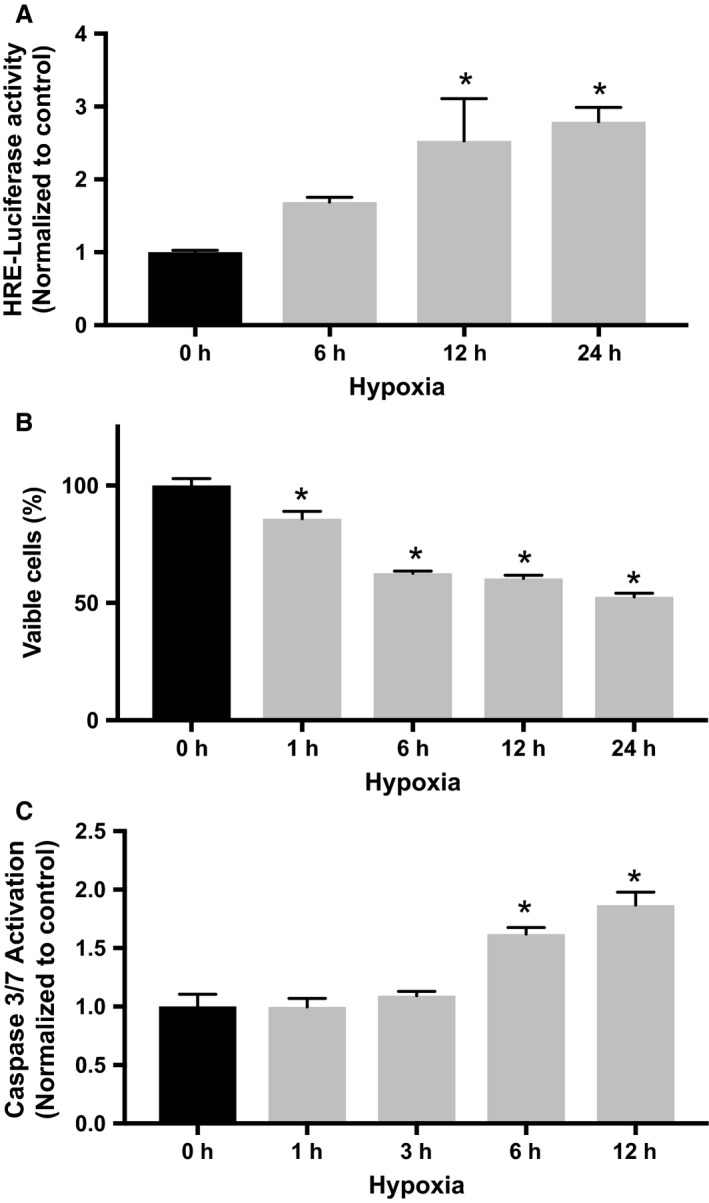
Validation of RNCM hypoxia model. (A) RNCMs transduced with AdV‐HRE luciferase were incubated in low‐oxygen conditions (<1% O_2_). HIF‐1 transcriptional activity, quantified by luciferase activity measure, increased as a function of incubation time spent in hypoxia. An “*” denotes a statistically significant difference compared with control. Data represent mean ± SEM (*n* = 4, ANOVA,* P* < 0.05, Tukey's test). (B) Cellular viability was assessed as described in Materials and Methods. A significant decrease in viability was detected after 1 hour of incubation in hypoxia. An “*” denotes a statistically significant difference compared with control. Data represent mean ± SEM (*n* = 4, ANOVA,* P* < 0.05, Tukey's test). (C) Apoptosis was quantified by measuring Caspase 3/7 activity. A significant increase in caspase activity was detected after 6 h of incubation in hypoxia. An “*” denotes a statistically significant difference compared with control. Data represent ± SEM (*n* = 3, ANOVA,* P* < 0.05, Tukey's test).

To ensure that our experimental design reproduced the physiological responses to hypoxia, we quantitated the magnitude and time course of HIF‐1 activation using a luciferase reporter assay. RNCM cultures were transduced with an adenovirus‐encoding luciferase under the control of the hypoxia response element (AdHRE‐luc). The intensity of the luminescent response is proportional to the amount of active HIF‐1 that interacts with the hypoxia response element (Lima et al. 2009). A statistically significant (~140%) increase in HIF‐1 activity was measured after 12 h of incubation in hypoxic conditions (Fig. 1A). HIF‐1 activity continued increasing throughout the 24 h of incubation in hypoxic conditions, indicating that the experimental model recapitulates the cellular responses to low oxygen.

To evaluate the effects of hypoxia on cultured RNCMs, cell viability was measured by quantifying metabolically active cells (Fig. 1B). There was a significant decrease after 1 hour of incubation in low‐oxygen conditions. There was no further decrease in metabolically active cells if incubation in low oxygen was continued beyond 12 h. The onset of apoptosis was determined by measuring Caspase 3/7 activation. A statistically significant (~60%) increase in protease activity indicating increased apoptosis was detected after 6 h of growth in a hypoxic environment (Fig. 1C). Incubation after 1 h in low oxygen also resulted in a loss of spontaneous contractile activity.

### Hypoxia reduces Cr transport in RNCM

**Figure 2:**
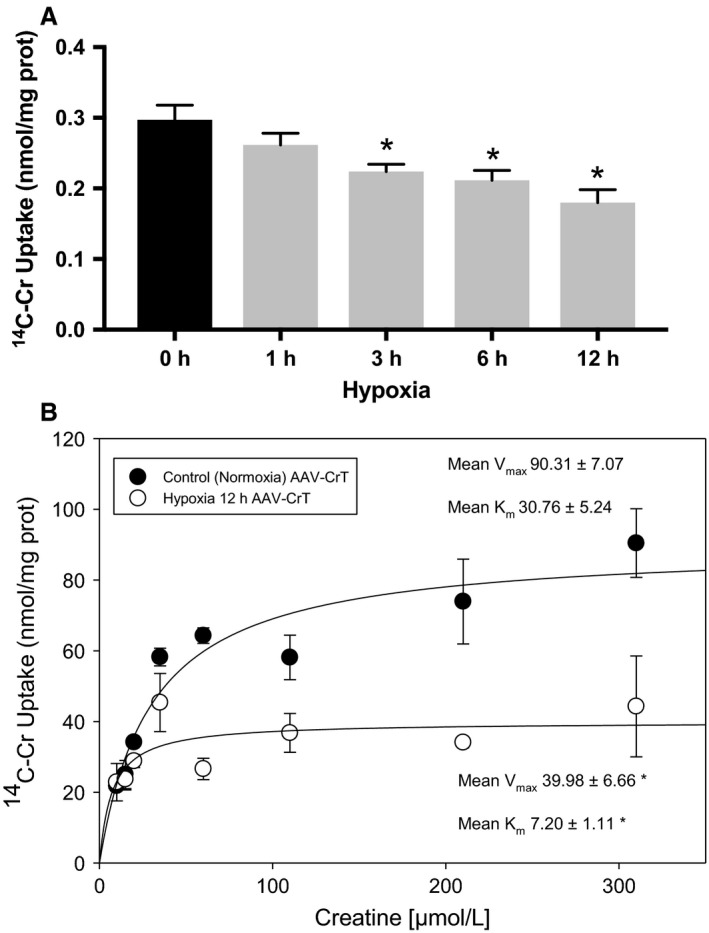
Hypoxia decreases Cr transport in RNCMs. (A) ^14^C‐Cr uptake was measured in RNCM cultures grown in a hypoxic atmosphere (<1% O_2_) for increasing periods of time. The data present the mean ± SEM of four independent ^14^C‐Cr uptake experiments. An “*” denotes a statistically significant decrease in transport compared with control cells (ANOVA,* P* < 0.05, Tukey's test). (B) A representative plot of Cr transport measured as a function of variable Cr concentrations (5–305 *μ*mol/L) is depicted. Kinetic analysis was performed as described in Materials and Methods for RNCMs in control conditions (filled circles) or after 12 h of hypoxia (hollow circles). Denoted *V*_max_ and *K*_m_ values are mean ± SEM. An “*” denotes a statistically significant difference compared with control normoxia values (*n* = 3, *T*‐test, *P* < 0.05).

^14^C‐Cr uptake was measured to determine the effects of hypoxia on Cr transport. Cr transport decreased significantly within the first 3 h of incubation in hypoxic conditions and continued to decline in a time‐dependent manner (Fig. 2A). The decline in Cr transport observed in RNCMs exposed to hypoxic conditions matched the observed decrease in viability (Fig. 1B) and antedated significant increases apoptosis (Fig. 1C).

RNCMs have low Cr transport capacity, which makes the determination of kinetic parameters such as *V*_max_ and *K*_m_ by ^14^C‐Cr uptake technically difficult. To better characterize the mechanisms mediating the decrease in Cr transport, we transduced RNCM cultures with an adeno‐associated virus (AAV) encoding the human isoform of CrT. We utilized a novel engineered AAV termed SASTG that conferred enhanced cardiac transduction (Piacentino et al. 2012) to deliver cDNA encoding CrT into cardiomyocytes. Transduced RNCMs exhibited Cr transport (Fig. 2B) with characteristic saturation kinetics and *V*_max_ of 90.31 ± 7.07 nmol/mg protein and a *K*_m_ of 30.76 ± 5.34 *μ*mol/L, which are well within the values reported in the literature (Loike et al. 1988; Dai et al. 1999; Darrabie et al. 2011). Characterization of kinetics of Cr transport in transduced RNCMs subjected to hypoxia for 12 h revealed a significant decrease in *V*_max_ (from 90.31 ± 7.07 to 30.76 ± 5.24 nmol/mg of protein) and *K*_m_ (from 30.76 ± 5.24–7.20 ± 1.11 *μ*mol/L) compared with controls (*n* = 3, *t*‐test *P* < 0.05).

### Pretreatment with AICAR prevents the decrease in Cr uptake induced by hypoxia

**Figure 3:**
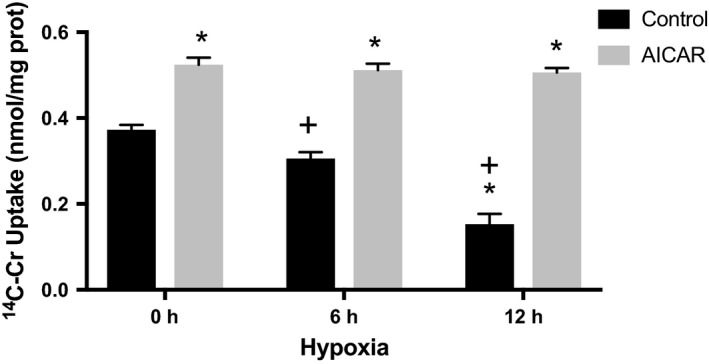
AICAR increases Cr transport in RNCMs subjected to hypoxia. RNCM cultures were pretreated with 0.5 mmol/L AICAR for 24 h before exposure to hypoxia for increasing periods of time and before measuring ^14^C‐Cr uptake. Data represent the mean ± SEM of three independent ^14^C‐Cr uptake experiments normalized to control (measured as nmoles/mg of protein). An “*” indicates a statistically significant difference in transport compared with controls (no AICAR and grown in normoxia) and a “+” indicates a statistically significant difference in transport compared to AICAR (grown in normoxia) (two‐way ANOVA,* P* < 0.05, Tukey's test).

Previously, we demonstrated that pharmacological activation of AMPK increases Cr transport in cardiomyocytes (Darrabie et al. 2011). Therefore, we tested the effect of pharmacological AMPK activation by 0.5 mmol/L AICAR beginning 24 h before exposure to hypoxia. Cr transport was significantly higher than that observed in controls (cultures exposed to hypoxia that did not receive AICAR) and remained elevated throughout the time course of the experiment (Fig. 3).

**Figure 4:**
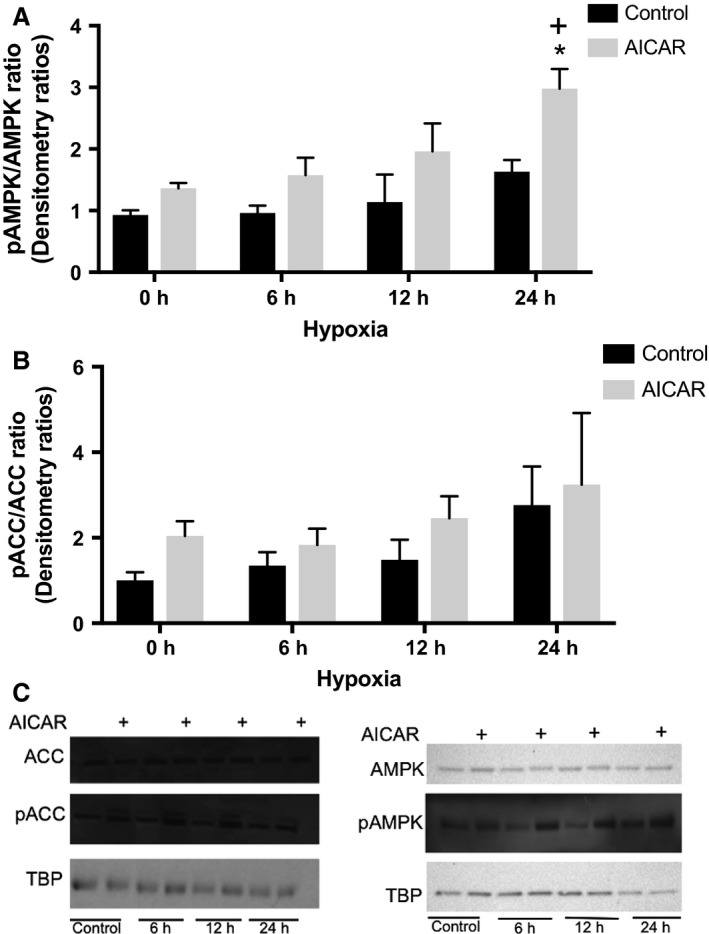
Quantification of AMPK activity in response to AICAR and/or hypoxia. (A) RNCM cultures were pretreated with or without 0.5 mmol/L AICAR for 24 h before exposure to hypoxia for the indicated time periods. The ratio of pAMPK/AMPK was determined using densitometry as described in Materials and Methods. Each bar represents means ± SEM of three independent experiments, normalized to control. An “*” denotes statistically significant difference compared with control at 0 h, and a “+” denotes statistically significant difference compared with AICAR at 0 h (two‐way ANOVA,* P* < 0.05, Tukey's test). (B) The pACC/ACC ratio was determined using densitometry as described in Materials and Methods. Each bar represents the mean ± SEM of three independent experiments (two‐way ANOVA,* P* = ns). (C) Representative immunoblots of pAMPK and pACC. TATA binding protein (TBP) was used as a loading control.

The activation of AMPK by AICAR and hypoxia was verified by densitometric quantification of the ratio of kinase phosphorylated at Thr172 (representing the active AMPK form, pAMPK) on immunoblots (Fig. 4C). The results indicate that sustained exposure to hypoxia and AICAR significantly increased the activation of AMPK by threefold after 24 h of incubation in low oxygen (Fig. 4A). Although not statistically significant, in the absence of AICAR, AMPK activation increased by 1.5‐fold after 24 h of incubation in hypoxic conditions. Active AMPK phosphorylates acetyl‐CoA carboxylase (ACC) and, therefore, the phosphorylated ACC (pACC), can serve as a biological reporter of AMPK activity (Darrabie et al. 2011). We measured the effects of incubation in medium supplemented with AICAR in culture incubated in low oxygen on the levels of pACC (Fig. 4C). Although not statistically significant, there appeared to be a progressive increase in the pACC/ACC ratio with incubation time in hypoxia, and the increase was accentuated by incubation with AICAR (Fig. 4B).

### Quantitation of ATP, Cr, and PCr

**Figure 5:**
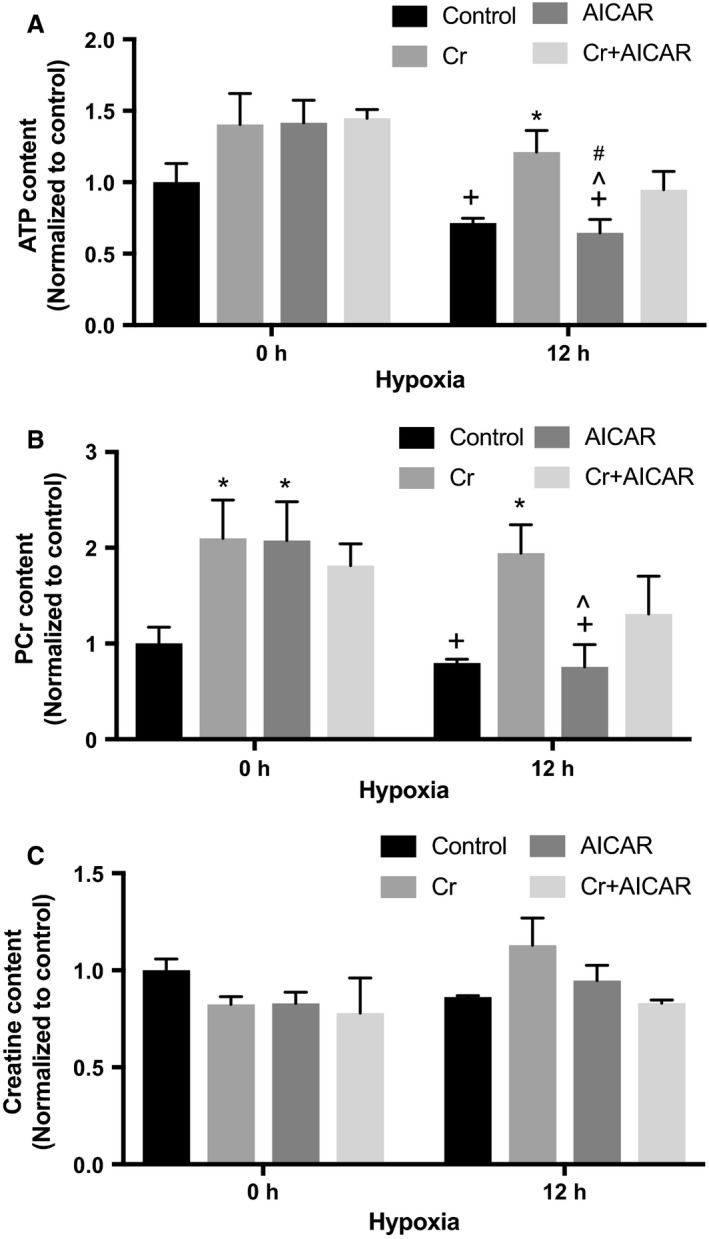
Quantification of ATP, PCr, and Cr. RNCM cultures were pretreated with or without 0.5 mmol/L AICAR, 1 mmol/L Cr, or both for 24 h before exposure to 12 h of hypoxia. (A) ATP content was measured as described in Materials and Methods. Each bar represents the mean ± SEM of three independent measurements, normalized to control values. An “*” denotes a statistically significant difference compared with controls at the same hypoxia time point, a “+” denotes a statistically significant difference compared with Cr at 0 h, a “^” denotes a statistically significant difference compared with AICAR at 0 h, and a “#” denotes a statistically significant difference compared with Cr + AICAR at 0 h (two‐way ANOVA,* P* < 0.05, Tukey's test). (B) PCr content was measured as described in Materials and Methods. Each bar represents the mean ± SEM of three independent measurements, normalized to control. An “*” denotes a statistically significant difference compared with Control at the same hypoxia time point, a “+” denotes a statistically significant difference compared with Cr at 0 h, and a “^” denotes a statistically significant difference compared with AICAR at 0 h (two‐way ANOVA,* P* < 0.05, Tukey's test). (C) Cr content was measured as described in Materials and Methods. Each bar represents the mean ± SEM of three independent measurements, normalized to control (two‐way ANOVA,* P* = ns).

The cellular content of ATP, Cr, and PCr was measured in RNCM cultures exposed to 12 h of hypoxia in control media or media supplemented with Cr, AICAR, or both, and compared to similar cultures grown in the presence of oxygen (Fig. 5). The results indicate supplementation with Cr, AICAR, or both increased the mean value of ATP content; however, this increase did not reach statistical significance in cultures grown under control oxygen conditions (Fig. 5A). Hypoxia did not significantly decrease ATP content in cultures grown in control media. However, RNCMs that were subjected to hypoxia and supplemented with Cr had elevated ATP content compared with nonsupplemented cells. Similarly, elevated ATP content was observed in hypoxia cultures treated with AICAR and Cr, but not when treated with AICAR alone. PCr content (Fig. 5B) was significantly elevated in cultures grown in control oxygen conditions and supplemented with Cr or AICAR when compared with nonsupplemented conditions. Exposure to hypoxia significantly decreased PCr content in RNCMs that received AICAR alone when compared to similar normoxic growth conditions. However, there was no significant decrease in PCr content in hypoxic cultures supplemented with Cr or Cr and AICAR combined. Cr content was also quantified in cultures supplemented with Cr, AICAR, or both following 12 h of hypoxia and compared with RNCMs grown in similar conditions and control oxygen levels. There were no statistically significant differences in Cr content among the different culture conditions (Fig. 5C).

### Cr supplementation augments HIF‐1 expression

**Figure 6:**
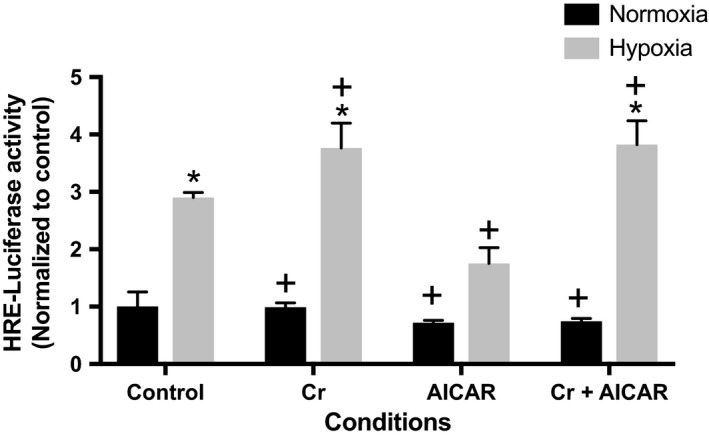
Creatine supplementation enhances HIF‐1 activity in RNCMs subjected to hypoxia. HIF‐1 function was quantified using a luciferase reporter in RNCM cultures supplemented with 0.5 mmol/L AICAR, 1 mmol/L Cr, or both for 24 h prior to 12 h incubation in hypoxic conditions, as described in Materials and Methods. Each bar represents the mean ± SEM of four independent experiments, normalized to control. An “*” denotes a statistically significant difference compared with control normoxia and a “+” denotes a statistically significant difference compared to control hypoxia (two‐way ANOVA,* P* < 0.05, Tukey's test).

We tested the effects of Cr supplementation (preincubation) alone or in combination with AICAR on HIF‐1 activation in cardiomyocytes exposed to 12 h of hypoxia (Fig. 6). Cr supplementation of hypoxic cells significantly increased HIF‐1 activity above that recorded in RNCMs exposed to hypoxia alone. Preincubation with AICAR had the opposite effect, significantly decreasing HIF‐1 activity. RNCM cultures that were preincubated with media supplemented with both Cr and AICAR also had significantly increased HIF‐1 activity compared with controls, of a magnitude similar to that recorded in cultures supplemented with Cr only.

## Discussion

Hypoxia has deleterious effects on energy metabolism, ultimately reducing ATP and PCr stores in tissues. In this study, we evaluated the effect of hypoxia on Cr transport in cardiomyocytes in culture. Cr transport decreased rapidly in RNCMs incubated in low‐oxygen conditions by as much as 35%. We evaluated the mechanisms responsible for the decrease in transport capacity by quantifying the effect of 12 h of incubation in low oxygen on *V*_max_ and *K*_m_ of transport. This time point was selected for our studies due to a significant activation of the hypoxia adaptive cellular responses, demonstrated by the HRE‐luciferase reporter assay at this time (Fig. 1A). RNCMs have inherently low Cr transport capacity, thus making accurate determination of *V*_max_ and *K*_m_ by ^14^C‐Cr uptake impossible. To overcome this limitation, we transduced RNCMs with an adeno‐associated virus that expresses the human CrT protein. Cr transport in transduced cells had characteristics similar to those reported previously in the literature for cardiomyocytes in culture (Darrabie et al. 2011). Kinetic analysis demonstrated a significant decrease in *V*_max_ (by 56%) and *K*_m_ (by 76%) compared with values measured in transduced cells grown under normoxic conditions. The decrease in *V*_max_, a functional measurement of active cell membrane transporters, indicates that the cell surface content of CrT protein is decreased, as previously demonstrated (Darrabie et al. 2011, 2012). A decrease in *K*_m_ indicates altered binding or release of Cr from the transporter, which decreases overall transport capacity. Taken together, these alterations in kinetic parameters suggest that hypoxia not only decreases the number of active transporters on the cell surface, but may also alter the structure of the CrT protein in such a way that the interactions with Cr during the substrate translocation cycle in the membrane are perturbed. A potential mechanism at play may be the appearance of intra‐ or intermolecular disulfide bonds that form under increased oxidative stress, which occurs in cells exposed to hypoxia. Indeed, rearrangements in disulfide bonds have been shown to underlie functional changes in other membrane transporters exposed to oxidative environments, such as the cardiac sodium–calcium exchanger (NCX1) (Santacruz‐Toloza et al. 2000).

AMPK is expressed at high levels in cardiac muscle but has low activity under normal physiological conditions, becoming more active during hypoxia (Borger et al. 2008; Omar et al. 2008). Together, HIF‐1 and AMPK activation are believed to be important for cellular survival (Wang et al. 2012). Previously, we demonstrated that pharmacological activation of AMPK increases Cr transport capacity in cultured cardiomyocytes (Darrabie et al. 2011). Here, we extend our observations to the effects of AMPK activation on Cr transport during hypoxia. Pharmacological AMPK activation not only preserved but actually increased Cr transport in RNCMs exposed to low oxygen concentrations (Fig. 3). AICAR also extended the time course of AMPK activation in RNCMs exposed to hypoxia. In the absence of pharmacological stimulation, significant AMPK activation was detected after 24 h of hypoxia in RNCMs (Fig. 4A and B). In these cardiomyocytes, the HIF‐1 mediated cellular mechanisms of adaptation to hypoxia developed before AMPK activity became statistically significant (Figs. 1A and 4A). HIF‐1 mediated responses were recorded as early as 6 h after the onset hypoxia, as demonstrated using a luciferase reporter under the control of HRE transcription control.

The activation pathways for AMPK and HIF‐1 are independent; however, a recent report demonstrates that in *Caenorhabditis elegans*, HIF‐1 and AMPK regulate each other reciprocally in response to oxidative stress. In this organism, HIF‐1 is phosphorylated by AMPK, decreasing its stability (Hwang et al. 2014). Such a mechanism is consistent with the reduced HIF‐1 activity we observed in cardiomyocytes supplemented with AICAR and incubated in hypoxic conditions (Fig. 6), where the boost in AMPK activity produced by AICAR treatment appears to dampen the early HIF‐1 response to hypoxia observed in RNCMs.

As mentioned previously, Cr supplementation has been reported to have a protective effect in both neurons, myoblasts, and cardiomyocytes in culture when subjected to hypoxic and increased oxidative stressors (Balestrino et al. 1999, 2002; Adcock et al. 2002; Caretti et al. 2010; Sartini et al. 2012). Given the role of Cr/PCr in maintaining ATP levels in the cell, we sought to quantify the effects of Cr supplementation, as well as that of AMPK activation alone or in combination with Cr supplementation on ATP, PCr, and Cr content. Cr cellular content did not change significantly with hypoxia or supplementations with Cr alone or in combination with AICAR. Cr is lost at a rate of 2% per 24 h by spontaneous conversion to creatinine (Wyss and Kaddurah‐Daouk 2000). Therefore, decreased Cr content due to diminished Cr transport capacity after incubation in hypoxic conditions may not be detectable in the time frame assayed. Cellular Cr may also be compartmentalized, and significant alterations in these intracellular Cr pools cannot be detected using a whole cell extract assay, or when testing relatively small cell numbers.. However, our results indicate that Cr supplementation of RNCMs significantly augments PCr levels under normal and hypoxic conditions.

Cr supplementation significantly increased ATP content in RNCMs subjected to hypoxia. In RNCMs incubated in control media and exposed to hypoxia, the decrease in ATP levels failed to reach significance when compared with control conditions. This is contrary to observations in intact hearts subjected to hypoxia, which very quickly become depleted of ATP and PCr. Adult cardiomyocytes depend on *β*‐oxidation of fatty acids to generate ATP, whereas the neonate cardiomyocytes have not developed *β*‐oxidation capabilities and use glycolysis as the main ATP generation pathway (Lopaschuk and Jaswal 2010). RNCMs are thus capable of maintaining constant ATP levels even when subjected to prolonged hypoxic stress. RNCMs spontaneously beat in culture; however, within 1 h of exposure to hypoxia, they stop contracting. Therefore, the overall consumption of ATP decreases as well, which would result in the ATP levels not changing significantly when compared with RNCMs grown in normoxia.

Further examination of the effect of Cr supplementation on the adaptive responses of RNCMs to hypoxia indicated that Cr supplementation initiated before hypoxia also significantly increased HIF‐1 activity compared with controls (hypoxia and no supplementation). Interestingly, when RNCM cultures were simultaneously supplemented with Cr and AICAR prior to hypoxia, HIF‐1 activity levels were significantly higher than those observed in control conditions, but similar in magnitude to those observed in cultures receiving Cr supplementation only. These observations suggest that Cr supplementation relieves the “inhibitory” effect that increased AMPK activity has on HIF‐1 function. Further studies to elucidate the mechanisms and signaling pathways mediating Cr supplementation‐enhanced HIF‐1 are warranted. An enhancement in the adaptive responses may also explain the protective effect of Cr supplementation reported in studies using mouse models of cerebral ischemia (Zhu et al. 2004) and stroke (Prass et al. 2007). The timing and duration of Cr supplementation in relation to the ischemic insult also needs to be carefully analyzed in order to fully realize the potential therapeutic value of Cr supplementation in a clinical setting.

In summary, we report for the first time that Cr transport is diminished within hours when cultured cardiomyocytes are exposed to hypoxia and that Cr supplementation significantly magnifies the physiological responses mediating adaptation and cell survival in low oxygen. This reduction in Cr transport capacity may contribute to the energy disturbances that characterize the ischemic heart. Cr supplementation increases ATP and PCr content in cardiomyocytes subjected to hypoxia. In addition to these increases, the ability to enhance the physiological response to oxidative stress, may also underlie some of the beneficial effects of Cr supplementation that have been previously reported, and merits further study.

## Conflict of Interest

None declared.
